# Comparison of nickel oxide nano and microparticles toxicity in rat liver: molecular, biochemical, and histopathological study

**DOI:** 10.1093/toxres/tfad062

**Published:** 2023-08-01

**Authors:** Caglar Adiguzel, Hatice Karaboduk, Fatma Gokce Apaydin, Suna Kalender, Yusuf Kalender

**Affiliations:** Faculty of Science, Department of Biology, Gazi University, Ankara 06500, Türkiye; Faculty of Science, Department of Biology, Gazi University, Ankara 06500, Türkiye; Faculty of Science, Department of Biology, Gazi University, Ankara 06500, Türkiye; Faculty of Gazi Education, Department of Science, Gazi University, Ankara 06500, Türkiye; Faculty of Science, Department of Biology, Gazi University, Ankara 06500, Türkiye

**Keywords:** liver, nickel oxide, nanoparticles, oxidative stress, apoptosis

## Abstract

The unique properties of nickel oxide nanoparticles distinguish it from classical nickel compounds, increasing its use in agriculture, industry, and many industrial areas. The aim of this study is to investigate the possible toxicity of nickel oxide and nickel oxide nanoparticles in the liver. For this purpose, Wistar rats were given nickel oxide and nickel oxide nanoparticles orally, intraperitoneally, and intravenously for 21 days. Liver organ weight, biochemical and hematological parameters, oxidative stress (malondialdehyde, catalase, superoxide dismutase, glutathione peroxidase, and glutathione S transferase), acetylcholinesterase activities, inflammation levels, apoptotic markers, and histopathological changes were evaluated comparatively. When the data obtained were examined in general, it was observed that nickel oxide nanoparticles caused more hepatotoxicity in liver tissue than nickel oxide in terms of oxidative stress parameters, apoptotic markers, inflammation indicators, and other parameters examined. The results suggest that toxicity induced by both nickel oxide and nickel oxide nanoparticles plays an important role in hepatocyte apoptosis.

## Introduction

Directly or indirectly involved in the production of many products in the nickel metal industry. Among the areas where nickel and its compounds are used most are steel production, aerospace and military applications, and battery and catalyst production.^1^ Since it is not a highly toxic metal, there are many nickels and its compounds produced for different purposes, and it has been stated that these compounds have different biological properties.^1^^,^^2^ Nickel oxide particles (NiO), one of these compounds are less toxic and less water-soluble than other nickel compounds. Since it is an important transition metal, it is frequently used in the production of gas sensors, magnetic materials, and batteries.^2^^,^^3^

With the development of nanotechnological applications, it has been seen that quite a variety of nanoparticles (NPs) have begun to be produced and scientific studies in this field are increasing.^4^ Thanks to the many specific properties of the designed NPs, their frequent use in the biomedical and pharmaceutical industries has led to the spread of many nano-based products to the environment, and as a result, they have become a direct or indirect environmental and public health problem.^5^^,^^6^ Studies have shown that in NP exposure, absorption occurs through the gastrointestinal tract and localizes in tissues including liver, kidney, spleen, and other organs, causing toxicity.^5^^,^^7^ Nickel oxide nanoparticles (NiONPs) are an important metal because of their very small size and large surface area, which is involved in the application of many products.^8^ The potential threat and toxicity of NiONPs, which have increased in use in recent years because of their commercial importance, on human health has also started to be shown in scientific publications.^9^ In scientific studies, it has been stated that exposure to NiONPs creates toxicity in living things, interacts with cell membranes and proteins, accumulates in tissues and organs, creates a cytotoxic effect, and also has a carcinogenic effect.^10^ The liver is among the soft tissues and is shown as a target organ in terms of xenobiotics toxicity. It has been shown that xenobiotics cause changes in liver enzymes such as alkaline phosphatase (ALP) and aspartate aminotransferase (AST) and cause histopathological changes.^11^ The toxicity of heavy metals in tissues and organs has been associated with the formation of free radicals.^12^ Under normal conditions, there is a balance between reactive oxygen species (ROS) and antioxidant enzyme activities. When this balance goes out of normal or shifts in favor of ROS, oxidative stress occurs in the body.^13^ Excessive production of ROS in the metabolic cycles of cells has been shown to cause serious damage to biomolecules, including membrane lipids.^14^ In a study, it was shown that NiONPs cause oxidative stress in the lungs and liver of rats, causing toxicity and serious damage.^15^ In another study, it was stated that nickel metal triggered oxidative stress in the rat liver and caused inflammation.^9^^,^^16^ The link or association of oxidative stress with apoptosis has been demonstrated many times.^17^ Apoptosis is programmed cell death that plays an active role in growth, development, and disease in advanced organisms.^13^ While apoptosis can occur in normal regulation, it can also occur in the case of oxidative stress-induced toxicity.^13^^,^^18^ Studies show that the pathways followed by apoptosis are divided into 2 as extrinsic and intrinsic (mitochondrial pathway), and it has been stated that the mitochondrial pathway is mostly followed.^13^^,^^19^^,^^20^ Both apoptotic pathways lead to activation of caspase-3 (Cas-3), leading to DNA fragmentation, cytoskeletal disruptions, and apoptotic cell formation.^21^ B-cell lymphoma-2 (Bcl-2) and Bcl-2-associated X protein (Bax) are mitochondria-derived cell apoptosis pathways.^22^ The anti-apoptotic protein Bcl-2 controls the membrane integrity of mitochondria and, when dominant, causes the cell to survive, whereas the pro-apoptotic protein Bax, when dominant, causes permeability in the mitochondrial outer membrane, causing the cell to die.^23^ The p53 protein is a transcription factor that plays an active role in DNA chain repair, cell cycle progression, and control, as well as the regulation of many cellular pathways such as apoptosis.^24^

Oral gavage application is one of the most frequently used experimental techniques among dosing studies in experimental animals, and it is the most basic application in obtaining clear results in enteric research.^25^ The intraperitoneal route of dosing is also a frequently used method and provides good and rapid absorption of large volumes of substances.^26^ Another route of administration is the intravenous route a good method for rapidly circulating highly concentrated or irritating substances.^27^

Within the scope of this study, Wistar male rats were selected and the histopathological, biochemical, and molecular changes in the liver of the rats were discussed comparatively by applying both microparticle and NP of NiO by oral, intraperitoneal, and intravenous administration routes.

## Materials and methods

### Reagents

NiONP (product number: NG04SO2802, CAS number: 1313-99-1) and NiO micropowder (product number: NG01OM1901, CAS number: 1313-99-1) from Nanografi Nano Technology (METU/Teknokent, Ankara, Türkiye) has been provided. NiONP are 10–40 nm in size, 99.55% of high purity, and NiO micropowders are 95% pure.

### Characterization of NiONP

X-ray analyses of NiONPs were performed on Bruker D8 Advance brand X-ray diffractometer (XRD) device at a scanning speed of 0.03° per second, CuKα (𝜆 = 1.5418 Å) beam, scanning in the range of 20–90°. Information on the surface morphology of NiONPs was obtained from Gazi University Faculty of Science, scanning electron microscope (SEM) JEOL JSM 6060 LV device.

### Animals and treatment schedule

Forty-two male Wistar rats were 250–300 g, purchased from the Laboratory Animals Breeding and Experimental Researches Center of Gazi University, and were used for this study. Animals in cage were housed at adequate temperature, fed diet, and water ad libitum. All experimental procedures were approved by Gazi University, Animal Experiments Local Ethics Committee (Protocol no: G.U. ET-21.033) and all experiments were performed in accordance with approved guidelines.

NiO and NiONP were prepared as a stock solution in physiological water, and sonicated for 30 s with an ultrasonicator before applying NiONP. NiO microparticle and NiONP were administered to experimental animals daily for 21 days at determined doses as oral gavage,^5^ intraperitoneally,^28^ and intravenously.^29^ In all, 42 rats to be used in this study were divided into 7 groups, 6 in each group.

Group I: Control group (distilled water).

Group II: NiO oral group (150 mg/kg bw per day).

Group III: NiO intraperitoneal (NiO IP) administration group (20 mg/kg bw per day).

Group IV: NiO intravenous (NiO IV) administration group (1 mg/kg bw per day).

Group V: NiONP oral group (150 mg/kg bw per day).

Group VI: NiONP intraperitoneal (NiONP IP) administration group (20 mg/kg bw per day).

Group VII: NiONP intravenous (NiONP IV) administration group (1 mg/kg bw per day).

On the completion of 21 days, rats were sacrificed by ketamine and xylazine combination, blood samples were taken from the hearts of rats, and liver tissues were removed for biochemical, cytopathological, histopathological, and molecular biology studies.

### Measurement of organ weights

After the anesthesia applied to the rats at the end of the 21-day application, the liver tissue was quickly removed. Livers were weighed in an automatic weighing device (AND GX-600, Japan) and weights were calculated in grams.

### Hematological examinations

Rat blood samples collected in EDTA tubes were analyzed for hemoglobin, leukocytes (WBC), hematocrit, erythrocyte (RBC), mean erythrocyte hemoglobin concentration (MCHC), mean erythrocyte hemoglobin (MCH), mean erythrocyte volume (MCV), red blood cell distribution width (RDW) (red blood cell distribution width can be reported statistically as coefficient of variation (RDW-CV) on the hemogram analyzer.

### Determination of biomarkers of oxidative stress

Liver tissues were homogenized using phosphate-buffered saline and centrifuged at +4°C (12,000 × rpm for 15 min). Enzyme-linked immunosorbent analysis (ELISA) commercial kits from BT LAB (Bioassay) Technology Laboratory, for rats according to the instructions of manufacturer’s, were used to determine malondialdehyde (MDA; Cat.No: E0156Ra) level and superoxide dismutase (SOD; Cat.No: E1444Ra), catalase (CAT; Cat.No: E0869Ra), glutathione peroxidase (GSH-PX; Cat.No: E1172Ra), glutathione S transferase (GST; Cat.No: E0513Ra) in the liver tissue of rats.

### Measurement of rat interleukin 1 beta concentration in liver tissues

After the liver tissues were homogenized with phosphate buffer, Rat Interleukin 1 Beta (IL-1β) ELISA Kit (Cat.No E0119Ra) from BT LAB was used and IL-1β monitoring was performed in rat liver tissue accordance with the manufacturer’s instructions.

### Acetylcholinesterase activity assay of liver tissue

Acetylcholinesterase (AChE) activity was specified from homogenized tissue spectrophotometrically using the method of Ellman et al.^30^

### Hepatic function assays

Lactate dehydrogenase (LDH), ALP, AST, and alanine aminotransferase (ALT) activities and levels of total cholesterol, triglyceride, total protein, and albumin of serum were designated using commercial kit and analyzed with an autoanalyzer.

### Real-time gene expression assay

Total RNA from liver tissue was extracted with QIAzol Lysis Reagent (Qiagen, Germany) and quantified by NanoDrop 2000. Total RNA was reverse transcribed to cDNA using RT2 First Strand Kit (Qiagen, Germany). qRT-PCR reactions were prepared from the diluted cDNA samples using primers for *Bax, Bcl2, Casp3, Tp53rk*, *Actin-beta* (*Actb; housekeeping gene*; Qiagen, Germany) and RT^2^ SYBR Green ROX FAST Mastermix (Qiagen, Germany) according to the instructions of manufacturer’s. mRNA expression experiments were quantified using the Rotor-Gene Q (QIAGEN, Germany) and duplicated for all samples to prevent errors because of manipulation. The Ct values were calculated using “the delta delta Ct” method, and the data were normalized using the values obtained in the control group’s average.

### Histopathology

Liver tissues were constant in 10% neutral formaldehyde. After routine tissue tracking procedures, tissues were embedded in paraffin blocks. Afterwards, paraffin-embedded tissue sample of 5–6-μm-thick sections was prepared and processed for hematoxylin and eosin staining (H&E). Sections (at least 10 slides) were viewed under a light microscope (Leica DM 100) to observe histopathological changes and photographed with a camera attached to the microscope (Leica DFC295).

Liver slides of all groups were examined and evaluated to show the degree of histopathological changes. Each slide was scored on a scale of no (0), mild (1), moderate (2), and severe (3) injury and used as an indicator for changes that occurred.

### Statistical analyses

SPSS program version 22 and GraphPad prism version 8 were used for all statistical analyses. Data were evaluated using ANOVA and Tukey tests. The obtained data were expressed as ±SD. *P* < 0.05 was considered significant.

## Results

### Characterization of NiONP

**Fig. 1:**
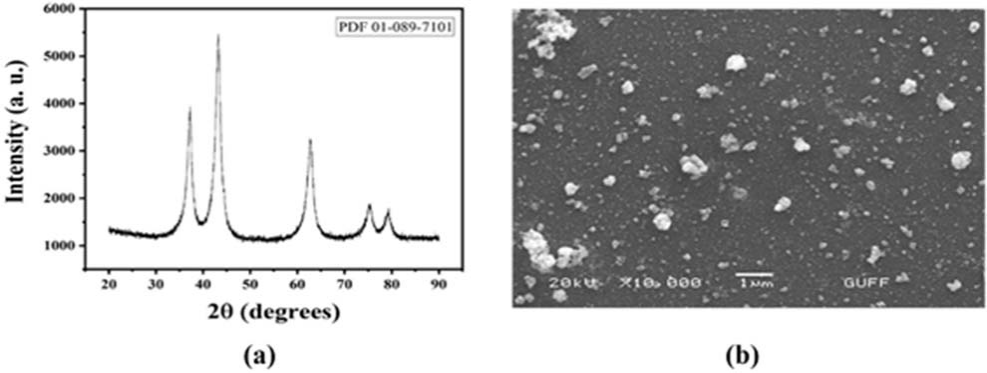
a) XRD diffractogram of NiONPs. b) SEM image of NiONPs.

The XRD plot is given in Fig. 1a to show the crystal phase of NiONPs. The intensity of the peaks obtained in the study is compatible with the density of JCPDS card No: 01-089-7101. The peaks at 37.10, 43.27, 62.69, 75.22, and 79.36 are associated with the 3D structure of NiONPs, and the sharpness of these peaks actually indicates that the NiONP is well crystallized. SEM image of NiONPs is given in Fig. 1b. In the examination made for NiONP, their shape structures are spherical and round, and their average size varies between 30 and 40 nanometers. In SEM examinations, NiONPs were visualized either singly or in small aggregates.

### Assessment of organ weights

No mortality was observed in experimental animals throughout the experiments. After 21 days, when the body and liver tissue weights of the control group and the rats administered oral, intraperitoneal, and intravenous NiO and NiONP were compared, no statistically significant change was observed. Diarrhea was observed throughout the experiments in rats treated with orally administered NiO and NiONP.

### Evaluation of hematological parameters

When the control group and NiO oral, NiO IP, NiO IV, NiONP oral, NiONP IP, and NiONP IV groups were compared, statistically significant difference was observed in hemoglobin, hematocrit, RBC, MCV, MCH, MCHC, RDW-CV, and WBC parameters (*P* < 0.05). When the treatment groups were compared among themselves, no statistically significant difference was observed in the hematological parameters of the rats (Table 1).

**Table 1:** Effects of exposure to NiO and NiONP on the hematological parameters of Wistar rat.

Parameters	Control	NiO oral	NiO IP	NiOIV	NiONP oral	NiONP IP	NiONP IV
Hemoglobin (g/dL)	15.45	13.08^a^	12.98^a^	12.37^a^	12.90^a^	12.61^a^	12.21^a^
Hematocrit (%)	49.16	46.23^a^	44.85^a^	44.61^a^	45.68^a^	44.66^a^	44.45^a^
RBC (M/mm^3^)	9.32	7.66^a^	7.65^a^	7.75^a^	7.34^a^	7.41^a^	7.14^a^
MCV (fL)	55.40	53.00^a^	52.93^a^	52.90^a^	52.91^a^	53.18^a^	51.85^a^
MCH (pg)	17.48	16.10^a^	16.31^a^	15.83^a^	16.03^a^	16.15^a^	15.70^a^
MCHC (g/dL)	32.40	30.96^a^	30.76^a^	30.80^a^	30.76^a^	30.55^a^	30.45^a^
RDW-CV (%)	17.45	14.78^a^	14.75^a^	14.61^a^	15.13^a^	14.50^a^	14.81^a^
WBC (K/mm^3^)	6.09	7.48^a^	7.70^a^	7.567^a^	7.53^a^	7.72^a^	8.16^a^

^a^Significant difference between control group and other groups. Significance at *P* < 0.05.

### Assessment of hepatic parameters and lipid profile

A statistically significant difference was observed between the control group and the NiO oral, NiO IP, NiO IV, NiONP oral, NiONP IP, and NiONP IV groups in terms of AST, ALT, ALP, LDH, triglyceride, total cholesterol, total protein, and albumin (*P* < 0.05). When the treatment groups were compared among themselves, no statistically significant difference was observed (Table 2).

**Table 2:** Effects of exposure to NiO and NiONP on the hepatic parameters and lipid profile of Wistar rat.

Parameters	Control	NiO oral	NiO IP	NiOIV	NiONP oral	NiONP IP	NiONP IV
AST (U/L)	150.00	191.00^a^	195.16^a^	197.66^a^	195.00^a^	198.33^a^	200.83^a^
ALT (U/L)	51.50	63.66^a^	64.16^a^	66.00^a^	64.66^a^	65.50^a^	65.83^a^
ALP (U/L)	105.00	123.83^a^	126.50^a^	127.33^a^	125.83^a^	126.33^a^	128.16^a^
LDH (U/L)	808.00	1250.00^a^	1260.83^a^	1266.16^a^	1254.33^a^	1263.33^a^	1272.33^a^
Triglyceride (mg/dI)	54.66	49.16	46.66	45.66	46.83	45.16	45.66
Total cholesterol (mg/dI)	47.50	65.33^a^	65.16^a^	68.16^a^	65.83^a^	66.16^a^	70.16^a^
Total protein (g/dI)	59.95	54.16^a^	53.18^a^	52.98^a^	53.51^a^	53.73^a^	53.28^a^
Albumin (g/dI)	35.80	31.85^a^	31.16^a^	30.60^a^	31.01^a^	30.13^a^	30.06^a^

^a^Significant difference between control group and other groups. Significance at *P* < 0.05.

### Evaluation of MDA levels and antioxidant enzyme activities

On the completion of the experiment, it was determined that the increase in the MDA levels of the liver tissues of the rats in NiO oral-, NiO IP-, NiO IV-, NiONP oral-, NiONP IP-treated groups was significantly compared with the control group. The MDA level in the NiONP IV-treated group was the highest among the 7 groups (*P* < 0.05). A significant decrease was observed in the applied groups when the CAT activity was compared with the control group. When the applied groups were compared among themselves, the decrease in the NiO IV- and NiONP IV-treated group was greater than in the other groups (*P* < 0.05). It was determined that the decrease in SOD activity in the NiO- and NiONP-treated groups was significant compared with the control, and the decrease in the NiONP IV-treated group was higher than the other groups in which NiO- and NiONP-treated groups (*P* < 0.05). GPx activity decreased significantly in the treated groups and was especially higher in the NiO IV- and NiONP IV-treated groups (*P* < 0.05). GST activity decreased to the control group in the applied groups, and the decrease especially in the NiONP IV-treated group was higher than the other treatment groups (*P* < 0.05; Table 3).

**Table 3:** Effects of exposure to NiO and NiONP on the MDA levels and antioxidant enzyme of Wistar rat.

Groups	Parameters				
	MDA (nmol/ml)	CAT (ng/ml)	SOD (ng/ml)	GPx (ng/ml)	GST (ng/ml)
Control	5.243	76.473	12.036	43.448	79.357
NiO oral	7.180^a^	59.104^a^	9.0235^a^	37.578^a^	50.102^a^
NiO IP	7.461^a^	59.129^a^	7.585^a^^,^^b^	35.439^a^	47.037^a^
NiO IV	7.636^a^	39.453^a^^,^^b^^,^^c^	6.809^a^^,^^b^	32.671^a^^,^^b^^,^^c^	38.683^a^
NiONP oral	7.370^a^	51.735^a^	8.277^a^^,^^b^	35.874^a^^,^^d^	41.776^a^
NiONP IP	7.500^a^	45.969^a^^,^^b^	7.298^a^^,^^b^^,^^e^	33.254^a^^,^^b^	39.546^a^
NiONP IV	7.888^a^^,^^b^	34.634^a^^,^^b^^,^^c^^,^^d^	6.553^a^^,^^b^^,^^c^^,^^e^	29.606^a^^,^^b^^,^^c^^,^^d^^,^^e^^,^^f^	33.492^a^^,^^b^^,^^c^

^a^Significant difference between control group and other groups.

^b^Significant difference between NiO oral group and other group.

^c^Significant difference between NiO IP group and other groups.

^d^Significant difference between NiO IV and other groups.

^e^Significant difference between NiONP oral and other groups.

^f^Significant difference between NiONP IP and other groups. Significance at *P* < 0.05.

### Evaluation of AChE activities

**Fig. 2:**
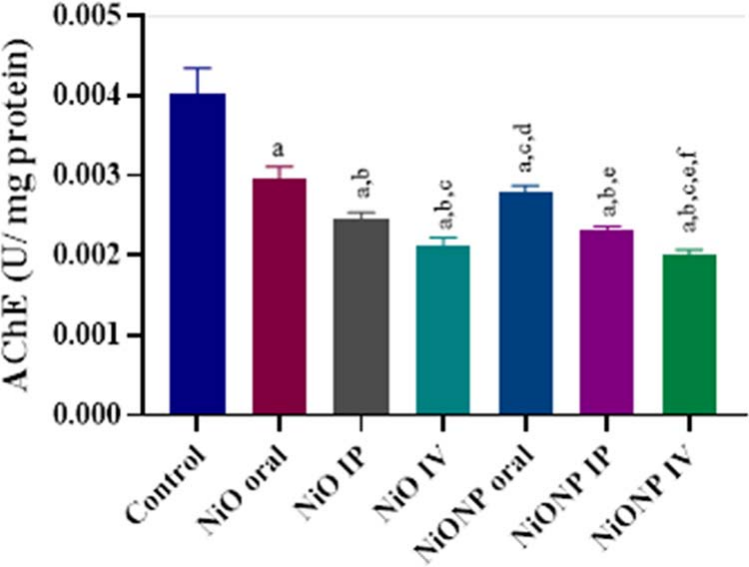
Effects of exposure to NiO and NiONP on the AChE (U/mg protein) activity of Wistar rat. ^a^Significant difference between control group and other groups. ^b^Significant difference between NiO oral group and other group. ^c^Significant difference between NiO IP group and other groups. ^d^Significant difference between NiO IV and other groups. ^e^Significant difference between NiONP oral and other groups. ^f^Significant difference between NiONP IP and other groups. Significance at *P* < 0.05.

There was a significant decrease in AChE activity in the treatment groups compared with the control group. When the application groups are compared, the decrease in AChE activity in the NiO IV group is higher than that of NiO IP and NiO oral. The decrease in AChE activity in NiONP IV group was higher than NiONP IP and NiONP oral. The highest decrease among the application groups was observed in the NiONP IV group (*P* < 0.05; Fig. 2).

### Evaluation of IL-1β activity

**Fig. 3:**
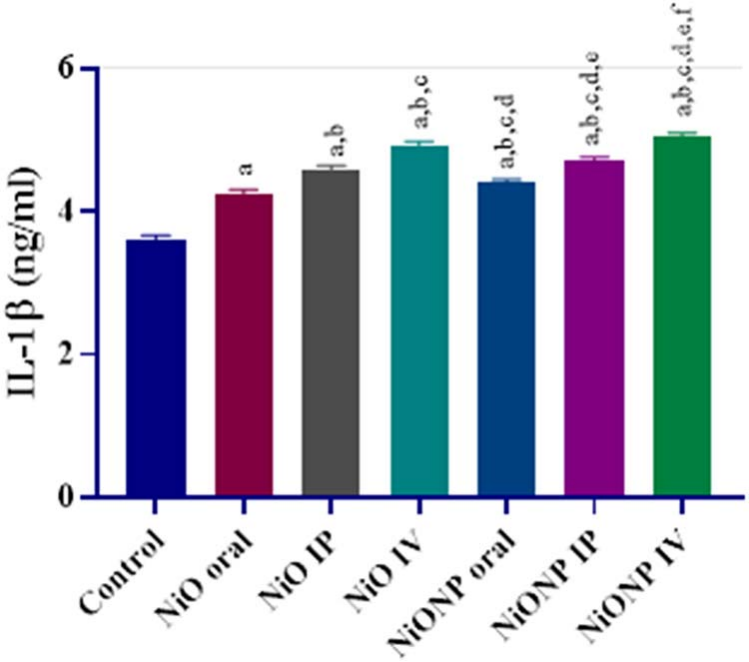
Effects of exposure to NiO and NiONP on the IL-1β activity of Wistar rat. ^a^Significant difference between control group and other groups. ^b^Significant difference between NiO oral group and other group. ^c^Significant difference between NiO IP group and other groups. ^d^Significant difference between NiO IV and other groups. ^e^Significant difference between NiONP oral and other groups. ^f^Significant difference between NiONP IP and other groups. Significance at *P* < 0.05.

IL-1β activity was investigated on the completion of experiment. It was observed that IL-1β activity increased in all applied groups compared with the control group. When the applied groups were evaluated among themselves, it was statistically seen that IL-1β activity in the NiO IV group was higher than NiO oral and NiO IP, whereas the IL-1β activity in the NP applied group was higher than NiONP oral and NiONP IP in the NiONP IV group. The highest increase in IL-1β level in the applied groups was in the NiONP IV group (*P* < 0.05; Fig. 3).

### Assessment of apoptosis status

**Fig. 4:**
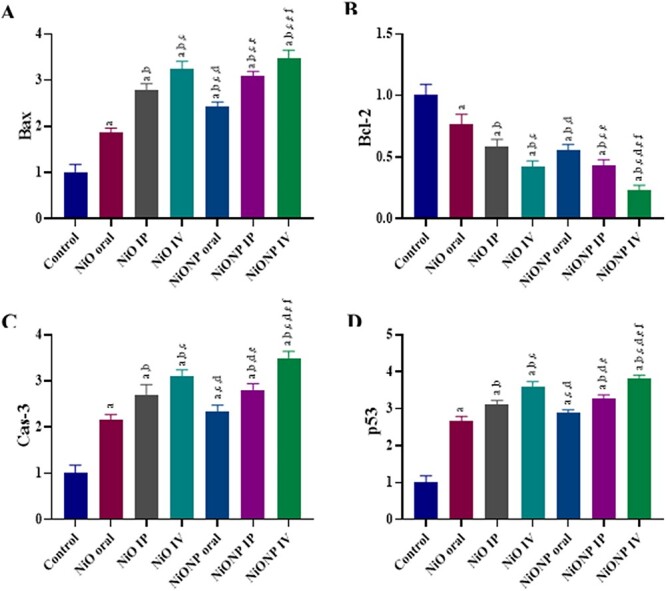
Effects of exposure to NiO and NiONP on apoptosis status in liver tissue of Wistar rats. a) NiO- and NiONP-induced Bax status in liver tissue. b) NiO- and NiONP-induced Bcl-2 status in liver tissue. c) NiO- and NiONP-induced Cas-3 status in liver tissue. d) NiO- and NiONP-induced p53 status in liver tissue. ^a^Significant difference between control group and other groups. ^b^Significant difference between NiO oral group and other group. ^c^Significant difference between NiO IP group and other groups. ^d^Significant difference between NiO IV and other groups. ^e^Significant difference between NiONP oral and other groups. ^f^Significant difference between NiONP IP and other groups. Significance at *P* < 0.05.

It was observed that NiO and NiONP administered to rats in different ways upregulated Bax, Cas-3, and p53 expressions in liver tissue, whereas downregulated Bcl-2 expression compared with the control group (Fig. 4a–d). When the application groups were evaluated among themselves, the NiO IV group upregulated Bax, Cas-3, and p53 expressions more, whereas it downregulated Bcl-2 more than the NiO oral and NiO IP groups (Fig. 4a–d). In NiONP groups, it was observed that while NiONP IV upregulated Bax, Cas-3, and p53 expressions more than other NP applied groups, it downregulated Bcl-2 expression more (Fig. 4a–d). NiONP IV was found to be the most effective group on Bax, Cas-3, p53, and Bcl-2 on liver tissue of rats (*P* < 0.05).

### Liver histopathological analysis

**Fig. 5:**
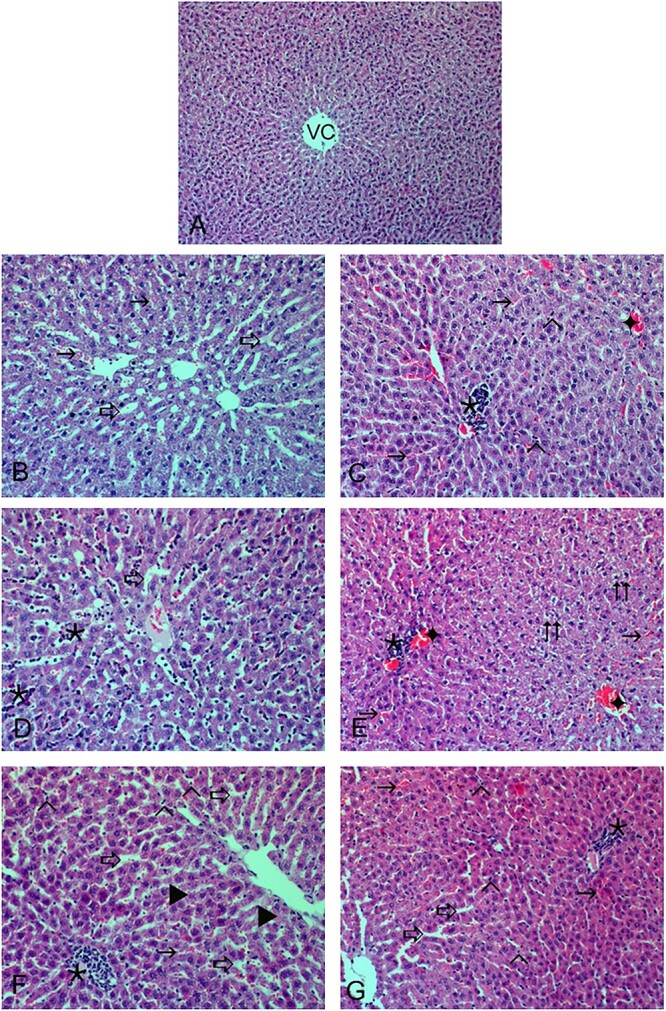
a) The appearance of liver tissues of control group rats under light microscope. CV: Central vein (VC) and hepatocytes appear normal, ×200. b) (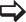) sinusoidal dilatation, (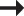) hemorrhage in NiO oral-treated group. c) (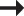) hemorrhage, (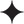) congestion, (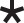) mononuclear cell infiltration, (^
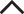
^) Kupffer cell proliferation in NiO IP-treated group. d) (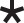) mononuclear cell infiltration, (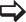) sinusoidal dilatation in NiO IV-treated group. e) (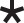) mononuclear cell infiltration, (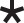) congestion, (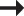) hemorrhage, and (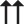) cellular degenerations in NiONP oral-treated group. f) (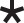) mononuclear cell infiltration, (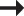) hemorrhage, (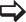) sinusoidal dilatation, (^
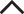
^) Kupffer cell proliferation, and (►) necrosis in NiONP IP-treated group. g) (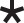) mononuclear cell infiltration, (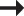) hemorrhage, (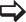) sinusoidal dilatation, (^
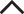
^) Kupffer cell proliferation in NiONP IV-treated group, ×200, H&E.

A normal histological structure of liver tissue was observed in rats of the control group (Fig. 5a). Sinusoidal dilatation and hemorrhage were detected in the liver tissue of rats treated with NiO oral groups (Fig. 5b). Mononuclear cell infiltration, hemorrhage, Kupffer cell proliferation, and congestion were observed in the liver tissue of rats in the NiO IP-treated groups (Fig. 5c). Sinusoidal dilatation and mononuclear cell infiltration were detected in the liver tissue of rats treated with NiO IV groups (Fig. 5d). Congestion, cellular degeneration, and mononuclear cell infiltration were observed and treated with NiONP oral groups (Fig. 5e). Mononuclear cell infiltration, hemorrhage, Kupffer cell proliferation, sinusoidal dilatation, and necrosis were observed in the liver tissue of rats in the NiONP IP-treated groups (Fig. 5f). Sinusoidal dilatation, hemorrhage, Kupffer cell proliferation, and mononuclear cell infiltration were detected in the NiONP IV groups (Fig. 5g). The histological changes are graded and summarized in Table 4.

**Table 4:** The scores of the histopathological changes in the liver sections of NiO and NiONP different administration exposure of rats.

Groups/parameters	Cell infiltration	Hemorrhage	Necrosis	Sinusoidal dilatation	Congestion	Kupffer cell proliferation
Control	0	0	0	0	0	0
NiO oral	0.50 ± 0.54	0.83 ± 0.40^a^	0	1.66 ± 0.51^a^	0.33 ± 0.51	0.50 ± 0.54
NiO IP	1.66 ± 0.81^a^^,^^b^	2.00 ± 0.63^a^^,^^b^	0	1.66 ± 0.51^a^	0.66 ± 0.51	1.66 ± 0.51^a^^,^^b^
NiO IV	1.83 ± 0.75^a^^,^^b^	1.83 ± 0.40^a^^,^^b^	0	2.16 ± 0.40^a^	0.66 ± 0.51	1.66 ± 0.51^a^^,^^b^
NiONP oral	2.33 ± 0.51^a^^,^^b^	2.16 ± 0.40^a^^,^^b^	0.33 ± 0.51	2.33 ± 0.51^a^	1.66 ± 0.51^a^^,b,c,^^d^	1.50±0.54^a^^,^^b^
NiONP IP	2.66 ± 0.51^a^^,^^b^	2.16 ± 0.40^a^^,^^b^	1.33 ± 0.51^a^^,b,c,d,^^e^	2.83 ± 0.40^a^^,b,^^c^	1.66 ± 0.51^a^^,b,c,^^d^	2.00±00^a^^,^^b^
NiONP IV	2.83 ± 0.40^a^^,^^b^^,^^c^	2.50 ± 0.63^a^^,^^b^	1.66 ± 0.51^a^^,b,c,d,^^e^	3.00±00^a^^,b,c,^^d^	1.66 ± 0.51^a^^,b,c,^^d^	2.00±00^a^^,^^b^

Data were presented as mean ± SD. *P* < 0.05.

A scale of 0–3: none change = 0; slight changes = 1; moderate changes = 2; and severe change = 3.

^a^Comparison of control and other groups.

^b^Comparison of NiO oral-treated group and other groups.

^c^Comparison of NiO IP-treated group and other groups.

^d^Comparison of NiO IV-treated group and other groups.

^e^Comparison of NiONP oral-treated group and other groups.

^f^Comparison of NiONP IP-treated group and other groups.

^g^Comparison of NiONP IV-treated group and other groups.

## Discussion

The fact that nanotechnology is used in many fields, especially in the industrial and biomedical fields, has brought it to a very important place. The use of NPs so frequently can be explained by research and experiments on mammalian organisms as a whole, including NP exposure, effects, toxicological risk assessments, dose–response relationships.^9^^,^^10^ The vital organ of xenobiotic metabolism and detoxification is the liver. It performs many functions such as regulation of protein and lipid synthesis, glycogen storage, and bile production.^6^ The liver has an organized and robust structure to remove the toxic substances accumulated in the body, but it is constantly exposed to environmental pollution and this causes the structure and functions of the liver to deteriorate.^31^^,^^32^ In the present study, toxicity caused by NiO and NiONP metals was compared with 3 different administration routes in terms of histological, molecular, biochemical, and hematological aspects. Statistical treatment of organ weights in experimental toxicology studies can be an important indicator in evaluating general health status and observing changes.^33^ In this study, it was observed that NiO and NiONP administration caused an increase in rat liver weight, but did not make a statistically significant difference with the control group. Regarding the weight change of NiO and NiONP on liver organ weights, Singh et al.^6^ and Yu et al.^9^ has similar studies that he has done before. In the 21-day results of the current study, it was observed that NiO and NiONPs can cause inflammation in the rat liver and longer exposure may increase inflammation. It has been shown in many studies that heavy metal exposures change hematological values.^14^ In this study, 21 days of NiO and NiONP administration caused a decrease in hemoglobin, hematocrit, RBC, MCV, MCH, MCHC, RDW-CV values and an increase in WBC values. WBC take an active role in maintaining body immunity and form the first line of defense. React strongly to infection or toxic chemicals.^34^ In this study, the increase in the number of WBCs in the applied groups is the result of the activation of the defense system and the activation of the immune cells.^10^^,^^35^ In addition, the increase in the number of WBC NiO and NiONP application created as an answer against the tissue destruction. The reduction in hemoglobin content and RBC count in the current study can be attributed to the ability of NiO and NiONPs to impair or pause the activities of hemoglobin synthesis and erythropoiesis.^4^^,^^36^ When the liver is chronically or acutely exposed to toxic agents, changes in its structure and functions may occur. Heavy metals can induce cell membrane damage and cytotoxic effects.^37^ When the plasma membrane of liver hepatocyte cells is damaged, serum enzymes such as ALT, ALP, AST, and LDH in the cytosol are released into the blood circulation. Therefore, these enzymes are reliable markers for assessing liver damage and have been studied by many researchers.^6^^,^^11^^,^^14^ In this study, when the ALT, AST, ALP, and LDH values were compared with the control group, there was a significant increase in the applied groups. Similar results are available in the studies of Abdulqadir and Aziz,^38^ Singh et al.,^6^ Atef Abdel et al.,^4^ Dumala et al.,^10^ Magaye et al.^29^ Toxic substances and drugs affect liver functions and hepatocyte structures, as well as alter lipid and protein metabolism.^39^ Albumin is a plasma protein synthesized by the liver and found in the blood. It takes an active role in binding drugs and chemicals.^40^ In this study, it was observed that albumin and total protein levels decreased in NiO and NiONP applied groups compared with the control. It can be said that NiO metal has a disruptive effect on protein metabolism in the liver. While there was a significant increase in the total cholesterol level in the NiO and NiONP groups, there was no significant change in the triglyceride level. It can be stated that NiO has a negative effect on liver lipid metabolism. Similar studies previously conducted by Pari and Prasath^16^ and Atef Abdel et al.^4^ have also been observed. When living beings are heavily exposed to toxic substance exposure, living metabolism generally leads to overproduction of ROS such as superoxide and hydroxyl. ROS are converted into non-harmful substances by enzymatic and nonenzymatic antioxidants in the body.^41^ There is a balance of antioxidants and oxidants in the body, and when this balance is disrupted in favor of the oxidant, oxidative stress occurs and as a result, the over-produced ROS results in irreversible damage.^42^ It has been shown by studies that NPs disrupt the intracellular oxidant-antioxidant balance, and it is stated that they create cytotoxicity in the cell and damage the protein, lipid, and DNA structure.^43^ The presence of oxidative stress, SOD, CAT, GPx, and GST antioxidant enzymes and the level of lipid peroxidation marker MDA have been investigated and shown many times in different tissues.^44^ In the current study, the MDA level was statistically higher in the NiO and NiONP applied groups than in the control group. Among the applied groups, only NiONP was significantly different from the NiO oral group, and there was no significant difference between the other applied groups. The significant increase in MDA level observed in the study may be related to the damage caused by NiO metal in cell membranes.

It is known that NPs cause damage to the antioxidant defense system by triggering the production of free radicals.^43^^,^^45^ In the current study, CAT, SOD, GPx, and GST activities decreased significantly in NiO and NiONP applied groups compared with the control group. While the most suppression of CAT activity was in NiO IV in NiO groups, it was in NiONP IV in NiONP groups. The greatest reduction for all groups was observed in the NiONP IV group. While the suppression of SOD activity in NiONP IV group was more than other applied groups, GPx activity was observed in NiO IV in NiO groups and in NiONP IV groups in NiONP groups. No significant difference was observed in the GST activity in the NiO and NiONP applied groups, except for the NiONP IV group. It was observed in our study that excessive amounts of free radicals caused by NiO and NiONPs lead to lipid peroxidation, leading to an increase in MDA level, and that antioxidant enzyme activities create toxicity by breaking the defense power. In studies on NiO and NiONP, both in the liver and in other organs of rats, the results of MDA levels and antioxidant enzyme activities were found to be consistent with the results of our study.^4^^,^^9^^,^^41^^,^^46^ AChE has an important role in acetylcholine metabolism in neurotransmission. AChE, located at cholinergic and neuromuscular synapses, is responsible for the breakdown of acetylcholine. AChE activity monitoring in cells is important in terms of neurotoxicity in reversible or irreversible poisoning caused by harmful chemicals.^10^^,^^44^ In the current study, it was observed that NiO and NiONP inhibited AChE activity in rat liver. In the NiO group, the inhibition in the NiO IV group was higher than that in the NiO oral and NiO IP groups, whereas the same was the case in the NP groups, and there was more inhibition in the NiONP IV group. When we look at the NiO and NiONP groups in general, the most inhibition of AChE activity was in the NiO IV and NiONP IV groups. Although AChE studies with NiO are not many in the literature, the results of studies on other organs are that NiO inhibits AChE activity, as in our study.^5^^,^^10^

Studies show that heavy metal exposures can lead to the accumulation of neutrophils, eosinophils and macrophages, leading to the activation of cytokines such as IL-1β.^47^ Interleukin 1 (IL-1) is a 17 kDa protein and is key to the initiation and progression of inflammation. Studies have shown that IL-1β plays an important role in the pathogenesis of inflammatory diseases.^48^ Under normal conditions, the cellular metabolism of cytokines is based on their synthesis and elimination by the liver, and an increase in IL-1 level has been observed in chronic liver diseases.^49^ In the current study, IL-1β level increased in all applied groups compared with the control group. The increase among the applied groups was mostly in the NiONP IV group. Studies have shown that NiONPs cause inflammatory effects and increase the effects of cytokines such as IL-1β.^50^ Increasing inflammatory cell flow in liver tissue with NiO and NiONP application is thought to be a response of the body to exogenous particles. Although there are not many studies on IL-1β in which NiO and NiONP are administered by different routes, it has been observed that there are increases in IL-1β levels in studies where NiONP is applied, which is consistent with our results.^8^^,^^51^ We used apoptotic markers to show the level of apoptosis in the toxicity caused by NiO and NiONP administration in liver tissue. NiO and NiONP application increased Bax, Cas-3, and p53 gene expression, whereas Bcl-2 gene expression was downregulated. The toxicity that occurs in mitochondria as a result of the interaction of ROS triggers oxidative stress, causing mitochondrial dysfunction, and plays an important role in cell death.^52^ One marker of hepatic dysfunction has also been shown to be apoptotic cell death.^53^ The toxicity created by NiO and NiONP administration causes the p53 gene to be upregulated, leading to the initiation of apoptosis signal in order to prevent the cell repair mechanism from spreading and to prevent the spread of damage or mutations.^52^^,^^54^ Downregulation of Bcl-2 and upregulation of Bax lead to the leakage of cytochrome c from the mitochondrial membrane. In this context, an increase in Bax and a decrease in Bcl-2 activate caspases.^55^ Cas-3 is a major point in apoptosis and is an irreversible process for the cell.^56^ In the current study, when apoptotic markers were examined, we found that NiONP IV had the most effect. Although the studies on NiO and NiONP with different application routes are not many in the literature, the results of the studies on NiO microparticles and NPs on different organs in the past show parallelism with our results.^22^^,^^52^^,^^57^ Evaluation of histopathological changes in tissues and organs is important in toxicological studies. In this study, toxic effect of NiO and NiONP was demonstrated by elevated serum biochemical parameters, the changes in oxidative stress parameters, as well as histopathological changes such as mononuclear cell infiltration, necrosis, sinusoidal dilatations, etc. These results led to serious changes in the general structure of liver tissue in response to NiO and NiONP. These histological changes may be because of increased MDA levels and reduced anti-oxidative enzyme activities in the liver tissue and it is exposed to many free radical effects in rats. Similar histopathological alterations in liver tissue were also reported by other researchers following xenobiotic.^44^^,^^58^^,^^59^ Toxicity studies with NiO microparticles and NPs in liver tissue have been done in the past and the results are similar to the current study.^4^^,^^5^^,^^6^^,^^9^

## Conclusion

As a result of our observations from the present work, we can write a few things. NiONPs were observed to be more toxic to liver tissue than NiO microparticles. Considering the dose rates and administration methods, it was determined that intravenous administration caused more toxicity and oxidative stress for the liver tissue. The increase in LPO level, decrease in antioxidant activities, changes in apoptotic markers, hepatic and biochemical results show that both NiO microparticle and NiONP pose a danger to the environment and public health, and care should be taken in their use.

## Data Availability

Data will be made available on request.

## References

[1] Henderson RG , Durando J, Oller AR, Merkel DJ, Maronne PA, Bates HK. Acute oral toxicity of nickel compounds. Regul Toxicol Pharmacol. 2012:62:425–432. 10.1016/j.yrtph.2012.02.002. 22333739

[2] Takahashi S , Oishi M, Takeda E, Kubota Y, Kikuchi T, Furuya K. Physicochernical characteristics and toxicity of nickel oxide particles calcined at different temperatures. Biol Trace Elem Res. 1999:69:161–173. 10.1007/BF02783867. 10433348

[3] El-Kemary M , Nagy N, El-Mehasseb I. Nickel oxide nanoparticles: synthesis and spectral studies of interactions with glucose. Mater Sci Semicond Process. 2013:16:1747–1752. 10.1016/j.mssp.2013.05.018.

[4] Atef Abdel MA , Mansour AB, Attia SA. The potential protective role of apigenin against oxidative damage induced by nickel oxide nanoparticles in liver and kidney of male Wistar rat, Rattus norvegicus. Environ Sci Pollut Res. 2021:28:27577–27592. 10.1007/s11356-021-12632-3. 33515148

[5] Dumala N , Mangalampalli B, Kalyan Kamal SS, Grover P. Repeated oral dose toxicity study of nickel oxide nanoparticles in Wistar rats: a histological and biochemical perspective. J Appl Toxicol. 2019:39(7):1012–1029. 10.1002/jat.3790. 30843265

[6] Singh M , Verma Y, Rana SVS. Hepatotoxicity induced by nickel nano and microparticles in male rat: a comparative study. Toxicol Environ Health Sci. 2021:13:251–260. 10.1007/s13530-021-00079-5.

[7] Lee IC , Ko JW, Park SH, Shin NR, Shin IS, Moon C, Kim JC. Comparative toxicity and biodistribution assessments in rats following subchronic oral exposure to copper nanoparticles and microparticles. Part Fibre Toxicol. 2016:13(1):56. 10.1186/s12989-016-0169-x. 27788687 PMC5084351

[8] Yang M , Chang X, Gao Q, Gong X, Zheng J, Liu H, Lil K, Zhan H, Wang X, Li S, et al. LncRNA MEG3 ameliorates NiO nanoparticles-induced pulmonary inflammatory damage via suppressing the p38 mitogen activated protein kinases pathway. Environ Toxicol. 2022:37:1058–1070. 10.1002/tox.23464. 35006638

[9] Yu S , Liu F, Wang C, Zhang J, Zhu A, Zou L, Han A, Li J, Chang X, Sun Y. Role of oxidative stress in liver toxicity induced by nickel oxide nanoparticles in rats. Mol Med Rep. 2018:17:3133–3139. 10.3892/mmr.2017.8226. 29257258

[10] Dumala N , Mangalampalli B, Srinivas S, Kamal K, Grover P. Biochemical alterations induced by nickel oxide nanoparticles in female Wistar albino rats after acute oral exposure. Biomarkers. 2018:23(1):33–43. 10.1080/1354750X.2017.1360943. 28748734

[11] Kalender S , Apaydin FG, Bas H, Kalender Y. Protective effects of sodium selenite on lead nitrate-induced hepatotoxicity in diabetic and non-diabetic rats. Environ Toxicol Pharmacol. 2015:40:568–574. 10.1016/j.etap.2015.08.011. 26339753

[12] Adıgüzel Ç , Kalender Y. Lead nitrate induced toxic effects on small intestine tissues in diabetic and non-diabetic rats: role of sodium selenite. GU J Sci. 2015:28(4):541–544.

[13] Kong L , Gao X, Zhu J, Cheng K, Tang M. Mechanisms involved in reproductive toxicity caused by nickel nanoparticle in female rats. Environ Toxicol. 2016:31(11):1674–1683. 10.1002/tox.22288. 27257140

[14] Uzunhisarcikli M , Aslanturk A, Kalender S, Apaydin FG, Bas H. Mercuric chloride induced hepatotoxic and hematologic changes in rats: the protective effects of sodium selenite and vitamin E. Toxicol Ind Health. 2016:32(9):1651–1662. 10.1177/0748233715572561. 25757480

[15] Liu F , Chang X, Tian M, Zhu A, Zou L, Han A, Su L, Li S, Sun Y. Nano NiO induced liver toxicity via activating the NF-κB signaling pathway in rats. Toxicol Res. 2017:6(2):242–250. 10.1039/c6tx00444j. PMC6060624 30090495

[16] Pari L , Prasath A. Efficacy of caffeic acid in preventing nickel induced oxidative damage in liver of rats. Chem Biol Interact. 2008:173:77–83. 10.1016/j.cbi.2008.02.010. 18405891

[17] Carraro U , Franceschi C. Apoptosis of skeletal and cardiac muscles and physical exercise. Aging. 1997:9:19–34. 10.1007/BF03340125. 9177583

[18] Aitken RJ , Gordon E, Harkiss D, Twigg JP, Milne P, Jennings Z, Irvine DS. Relative impact of oxidative stress on the functional competence and genomic integrity of human spermatozoa. Biol Reprod. 1998:59:1037–1046. 10.1095/biolreprod59.5.1037. 9780307

[19] Zhao Y , Xu L, Qiao Z, Gao L, Ding S, Ying X, Su Y, Lin N, He B, Pu J. YiXin-Shu, a ShengMai-San-based traditional Chinese medicine formula, attenuates myocardial ischemia/reperfusion injury by suppressing mitochondrial mediated apoptosis and upregulating liver-X-receptor alpha. Sci Rep. 2016:6(23025):1–13. 10.1038/srep23025. 26964694 PMC4786861

[20] Tang D , Lotze MT, Kang R, Zeh HJ. Apoptosis promotes early tumorigenesis. Oncogene. 2011:30:1851–1854. 10.1038/onc.2010.573. 21151175

[21] Shojaie L , Iorga A, Dara L. Cell death in liver diseases: a review. Int J Mol Sci. 2020:21(24):1–47. 10.3390/ijms21249682 PMC7766597 33353156

[22] Kong L , Hu W, Lu C, Cheng K, Tang M. Mechanisms underlying nickel nanoparticle induced reproductive toxicity and chemo-protective effects of vitamin C in male rats. Chemosphere. 2019:218:259–265. 10.1016/j.chemosphere.2018.11.128. 30472609

[23] Liu L , Zhou Y, Dai D, Xia H, Zhao K, Zhang J. Protective effects of Kangxian ruangan capsule against nonalcoholic fatty liver disease fibrosis in rats induced by MCDdiet. Biomed Pharmacother. 2018:108:424–434. 10.1016/j.biopha.2018.06.134. 30236852

[24] Koçak N , Yıldırım İH, Yıldırım SC. p53 ve p53 gen ailesi üyeleri olan p63 ve p73’ün hücresel işlevleri. Dicle Med J. 2011:38(4):530–535. 10.5798/diclemedj.0921.2011.04.0083.

[25] Jones CP , Boyd KL, Wallace JM. Evaluation of mice undergoing serial oral gavage while awake or anesthetized. J Am Assoc Lab Anim Sci. 2016:55(6):805–810. 27931321 PMC5113884

[26] Lambert LJ , Muzumdar MD, Rideout WM, Jacks T. Basic mouse methods for clinician researchers: harnessing the mouse for biomedical research. Academic Press. 2017:291–312. 10.1016/B978-0-12-803077-6.00014-X.

[27] Hedrich H . The laboratory mouse. Cambridge: Academic Press; 2004 pp. 527–532

[28] Marzban A , Seyedalipour B, Mianabady M, Taravati A, Hoseini SM. Biochemical, toxicological, and histopathological outcome in rat brain following treatment with NiO and NiO nanoparticles. Biol Trace Elem Res. 2020:196:528–536. 10.1007/s12011-019-01941-x. 31902099

[29] Magaye RR , Yue X, Zou B, Shi H, Yu H, Liu K, Lin X, Xu J, Yang C, Wu A, et al. Acute toxicity of nickel nanoparticles in rats after intravenous injection. Int J Nanomedicine. 2014:9:1393–1402. 10.2147/IJN.S56212. 24648736 PMC3958504

[30] Ellman GL , Courtney KD, Andres V, Featherstone RM. A new and rapid colorimetric determination of acetylcholinesterase activity. Biochem Pharmacol. 1961:7:88–95. 13726518 10.1016/0006-2952(61)90145-9

[31] Akin AT , Öztürk E, Kaymak E, Karabulut D, Yakan B. Therapeutic effects of thymoquinone in doxorubicin-induced hepatotoxicity via oxidative stress, inflammation and apoptosis. Anat Histol Embryol. 2021:50:908–917. 10.1111/ahe.12735. 34494664

[32] Karaboduk H , Kalender Y. The effects of lead nitrate and mercury chloride on rat liver tissue. Fresenius Environ Bull. 2021:30(3):2368–2379.

[33] Ogutcu A , Uzunhisarcikli M, Kalender S, Durak D, Bayrakdar F, Kalender Y. The effects of organophosphate insecticide diazinon on malondialdehyde levels and myocardial cells in rat heart tissue and protective role of vitamin E. Pestic Biochem Physiol. 2006:86:93–98. 10.1016/j.pestbp.2006.01.010.

[34] Baker AA , Alkshab AA, Ismail H. Kh, effect of silver nanoparticles on some blood parameters in rats. Iraqi J Vet Sci. 2020:34(2):389–395. 10.33899/ijvs.2019.126116.1243.

[35] Gui S , Zhang Z, Zheng L, Cui Y, Liu X, Li N, Sang X, Sun Q, Gao G, Cheng Z, et al. Molecular mechanism of kidney injury of mice caused by exposure to titanium dioxide nanoparticles. J Hazard Mater. 2011:195:365–370. 10.1016/j.jhazmat.2011.08.055. 21907489

[36] Morsy GM , El-Ala KSA, Ali AA. Studies on fate and toxicity of nanoalumina in male albino rats: some haematological, biochemical and histological aspects. Toxicol Ind Health. 2016:32(4):634–655. 10.1177/0748233713504022. 24215066

[37] Anuradha R , Krishnamoorthy P. Impact of *Pongamia pinnata* extract on lead acetate mediated toxicity in rat liver. Int J Pharmtech Res. 2012:4:878–882.

[38] Abdulqadir SZ , Aziz FM. Hepatotoxicity of nickel nanoparticles in rats. Indian J Anim Res. 2019:0367–6722. 10.18805/ijar.B-1100.

[39] Mohamed ET , Mahran HA, Mahmoud MS. Hepato ameliorative effect of *Azadirachta indica* leaves extract against mercuric chloride environmental pollution. J Am Sci. 2010:6(9):735–751.

[40] Apaydin FG , Baş H, Kalender S, Kalender Y. Bendiocarb induced histopathological and biochemical alterations in rat liver and preventive role of vitamins C and E. Environ Toxicol Paharmacol. 2017:49:148–155. 10.1016/j.etap.2016.11.018. 28013143

[41] Noshy PA , Khalaf AAA, Ibrahim MA, Mekkawy AM, Abdelrahman RE, Farghali A, Tammam AAE, Zaki AR. Alterations in reproductive parameters and steroid biosynthesis induced by nickel oxide nanoparticles in male rats: the ameliorative effect of hesperidin. Toxicology. 2022:473:153208. 10.1016/j.tox.2022.153208. 35569531

[42] Wu Y , Kong L. Advance on toxicity of metal nickel nanoparticles. Environ Geochem Health. 2020:42:2277–2286. 10.1007/s10653-019-00491-4. 31894452

[43] Singh M , Verma Y, Rana SVS. Nephrotoxicity of nickel nano and microparticles in rat- a comparative, time dependent study with special reference to antioxidant defence system. Inorg Nano-Met Chem. 2022:52(9):1335–1344. 10.1080/24701556.2022.2048307.

[44] Baş H , Apaydın FG, Kalender S, Kalender Y. Lead nitrate and cadmium chloride induced hepatotoxicity and nephrotoxicity: protective effects of sesamol on biochemical indices and pathological changes. Food Chem. 2021:45(7):e13769. 10.1111/jfbc.13769. 34021611

[45] Manke A , Wang L, Rojanasakul Y. Mechanisms of nanoparticle-induced oxidative stress and toxicity. Biomed Res Int. 2013:ID 942916. 10.1155/2013/942916. PMC3762079 24027766

[46] Zhu A , Chang X, Sun Y, Zou L, Su L, Sun Y, Li S, Liu S, Sun Y, Zhou H, et al. Role of oxidative stress and inflammatory response in subchronic pulmonary toxicity induced by nano nickel oxide in rats. J Nanosci Nanotechnol. 2017:17:1753–1761. 10.1166/jnn.2017.12849.

[47] Gillespie AA , Kang GS, Elder A, Gelein R, Chen L, Moreira AL, Koberstein J, Tchou-Wong KM, Gordon T, Chen LC. Pulmonary response after exposure to inhaled nickel hydroxide nanoparticles: short and long-term studies in mice. Nanotoxicology. 2010:4(1):106–119. 10.3109/17435390903470101. 20730025 PMC2922767

[48] Cano-Cano F , Jaramillo LG, Garcia PR, Arroba AI, Diosdado MA. IL-1β implications in type 1 diabetes mellitus progression: systematic review and meta-analysis. J Clin Med. 2022:11:1303. 10.3390/jcm11051303. 35268394 PMC8910979

[49] Sayed S , Alotaibi SS, El-Shehawi AM, Hassan MM, Shukry M, Alkafafy M, Soliman MM. The anti-inflammatory, anti-apoptotic, and antioxidant effects of a pomegranate-peel extract against acrylamide-induced hepatotoxicity in rats. Life. 2022:12:224. 10.3390/life12020224. 35207511 PMC8878900

[50] Morimoto Y , Ogami A, Todoroki M, Yamamoto M, Murakami M, Hirohashi M, Oyabu T, Myojo T, Nishi KI, Kadoya C, et al. Expression of inflammation-related cytokines following intratracheal instillation of nickel oxide nanoparticles. Nanotoxicology. 2010:4(2):161–176. 10.3109/17435390903518479. 20795893

[51] Cao Z , Fang Y, Lu Y, Qian F, Ma Q, He M, Pi H, Yu Z, Zhou Z. Exposure to nickel oxide nanoparticles induces pulmonary inflammation through NLRP3 inflammasome activation, in rats. Int J Nanomedicine. 2016:11:3331–3346. 10.2147/IJN.S106912. 27524893 PMC4965228

[52] Saquib Q , Attia SM, Ansari SM, Al-Salim A, Mohammad F, Alatar AA, Musarrate J, Zhang X, Al-Khedhairy AA. p53, MAPKAPK-2 and caspases regulate nickel oxide nanoparticles induce cell death and cytogenetic anomalies in rats. Int J Biol Macromol. 2017:105:228–237. 10.1016/j.ijbiomac.2017.07.032. 28690165

[53] Dutordoir MR , Bates DAA. Activation of apoptosis signalling pathways by reactive oxygen species. Biochim Biophys Acta Mol Cell Res. 2016:1863(12):2977–2992. 10.1016/j.bbamcr.2016.09.012. 27646922

[54] Meyer K , Rajanahalli P, Ahamed M, Rowe JJ, Hong Y. ZnO nanoparticles induce apoptosis in human dermal fibroblasts via p53 and p38 pathways. Toxicol In Vitro. 2011:25:1721–1726. 10.1016/j.tiv.2011.08.011. 21903158

[55] Noor KK , Ijaz MU, Ehsan N, Tahir A, Yeni DK, Zihad SMNK, Uddin SJ, Ashraf A, Gandara JS. Hepatoprotective role of vitexin against cadmium-induced liver damage in male rats: a biochemical, inflammatory, apoptotic and histopathological investigation. Biomed Pharmacother. 2022:150:112934. 10.1016/j.biopha.2022.112934. 35421786

[56] Green DR , Amarante-Mendes GP. The point of no return: mitochondria, caspases, and the commitment to cell death. Results Probl Cell Differ. 1998:24:45–61. 10.1007/978-3-540-69185-3_3. 9949831

[57] Chang X , Liu F, Tian M, Zhao H, Han A, Sun Y. Nickel oxide nanoparticles induce hepatocyte apoptosis via activating endoplasmic reticulum stress pathways in rats. Environ Toxicol. 2017:32:2492–2499. 10.1002/tox.22492. 28945320

[58] Apaydın FG , Kalender S, Kalender Y. Subacute exposure to dimethoate induces hepatotoxic and nephrotoxic effects on male rats: ameliorative effects of ferulic acid. Indian J Exp Biol. 2023:61:51–28. 10.56042/ijeb.v61i01.49688.

[59] Uzunhisarcikli M , Apaydin FG, Bas H, Kalender Y. Hepatoprotective effects of quercetin and curcumin against fipronil-induced hepatic injury in rats. Fresenius Environ Bull. 2021:30:9309–9321.

